# A Sensitive Chemotaxis Assay Using a Novel Microfluidic Device

**DOI:** 10.1155/2013/373569

**Published:** 2013-09-14

**Authors:** Chen Zhang, Sunyoung Jang, Ovid C. Amadi, Koichi Shimizu, Richard T. Lee, Richard N. Mitchell

**Affiliations:** ^1^Department of Pathology, Brigham and Women's Hospital and Harvard Medical School, Boston, MA 02115, USA; ^2^Department of Hepatobiliary Surgery, Union Hospital, Tongji Medical College of Huazhong University of Science and Technology, Wuhan, Hubei, China; ^3^Cardiovascular Division, Brigham and Women's Hospital and Harvard Medical School, Boston, MA 02115, USA; ^4^Harvard-MIT Division of Health Sciences and Technology, Massachusetts Institute of Technology, Cambridge, MA 02115, USA

## Abstract

Existing chemotaxis assays do not generate stable chemotactic gradients and thus—over time—functionally measure only nonspecific random motion (*chemokinesis*). In comparison, microfluidic technology has the capacity to generate a tightly controlled microenvironment that can be stably maintained for extended periods of time and is, therefore, amenable to adaptation for assaying chemotaxis. We describe here a novel microfluidic device for sensitive assay of cellular migration and show its application for evaluating the chemotaxis of smooth muscle cells in a chemokine gradient.

## 1. Introduction

Directed cell migration plays a critical role in inflammatory disorders, vascular disease, wound healing, and tumor metastasis [1–5]. A number of *in vitro* approaches have been developed to quantify cell migration, including closure of monolayer wounds (“scratch assay”) and the Boyden chamber [6–8]. However, these current methods are relatively insensitive. Moreover, such approaches may actually measure only *chemokinesis*, that is, increased random motion, rather than *chemotaxis*—directed cell migration.

Indeed, the utility of the scratch assay and Boyden chamber transwells [9, 10] is limited by their methodologic design.

For the scratch assay, a defined “wound” (scratch) is made in a confluent cell monolayer; cells at the edges of the defect then progressively fill in the void, and the time to restored confluence is quantified. Faster repair of the scratch has been interpreted to reflect enhanced “chemotaxis” due to cell manipulation or the nature of soluble agents added to the medium. While the experiment is conceptually straightforward and technically easy to perform, the cells are bathed in a uniform concentration of agent, and there is no chemoattractant concentration gradient during the entire experiment; the movement that fills in the gap therefore represents only a “random walk.” Thus, the “migration” in a scratch assay is largely a function of only increased chemokinesis. Moreover, as typically performed—especially over several hours of a prolonged assay—the closing of a scratch “wound” also likely includes a substantial component of cell proliferation.

The other most common method to measure “chemotaxis” involves the use of Boyden chamber transwells. Different concentrations of specific chemokines are placed in the lower compartment of the device, while the cells to be evaluated are incubated in the upper insert; a microporous membrane separates the two chambers and forms a support for cell growth and a partial barrier for migration. Cells that are counted at the lower face of the membrane, or that accumulate in the lower chamber, are typically assessed at a single fixed end point. This assay has the advantage of an apparent directed migration, that is, from upper to lower chamber but is still problematic. First, there is no “gradient” of chemokines from top to bottom but rather only a single step function from low to high concentrations. Second, there is no way to sustain the chemokine differential from the top to bottom chambers [11]. Initially, the chemokine concentration in the lower compartment is greater but within minutes to hours, the concentrations equalize due to diffusion, at which point specific chemotaxis ceases. Instead, long-term measurements more likely reflect a significant element of chemokinesis. Moreover, once cells fall through the membrane and into the lower chamber, there is no opportunity for reverse migration. Finally, the Boyden chamber is also limited because cell counting requires termination of the experiment; a time course therefore requires multiple devices.

**Figure 1:**
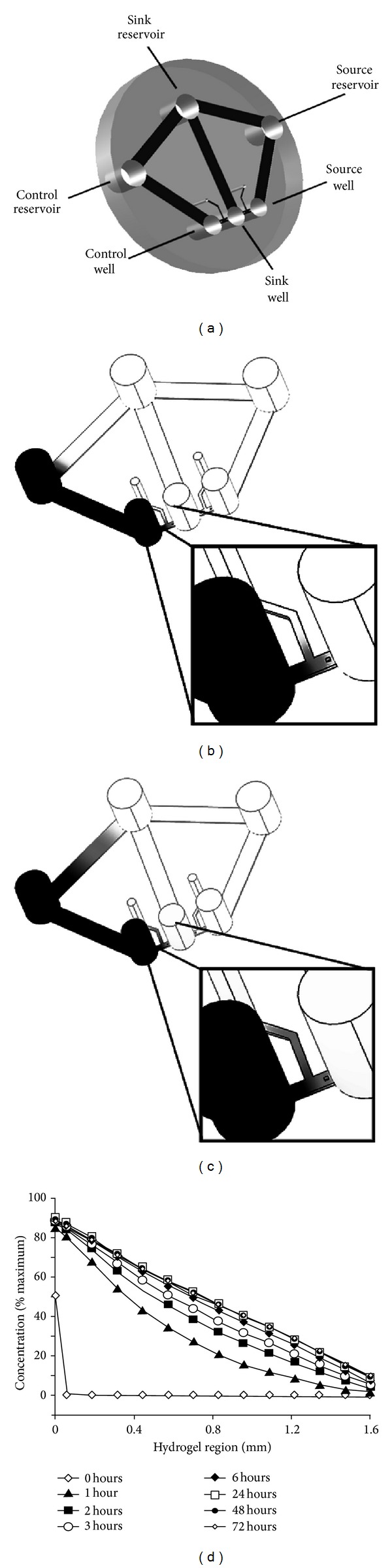
Device design and stability of concentration gradients as a function of time. (a) The lower 3 wells are cell and/or source wells for chemokines, and the upper 3 wells serve as reservoirs to maintain equivalent pressures in the lower wells. Depending on experimental design, the side wells or the central well can serve as source wells; cells in a central well can migrate toward different chemokine stimuli on either side, or different cell populations in the side wells can migrate towards a central chemokine stimulus. (b, c) Concentration gradients are shown schematically after the addition of a low molecular weight fluorescent dye indicator to the source well and source reservoir, at either 2 hours (b) or 72 hours (c). (d) Graphical display of concentration gradients from 0 hour through 72 h after fluorescent dye addition to source well and reservoir.

Microfluidic technology can overcome these limitations by generating a long-term stable and controllable gradient of soluble factors that can be continuously monitored over time [12–16]. Using cells that have been treated to block proliferation, such a device allows a true ongoing assessment of chemotaxis versus chemokinesis.  Figure 1 depicts such a device where the source and sink concentrations are maintained by creating corresponding wells whose volumes are large relative to the diffusive flux through the connecting hydrogel channel [17]. At steady state, a linear concentration gradient forms between these two wells. Although interstitial flow through the hydrogel region can disrupt the gradient if the hydrostatic pressures in the two wells are not equal, pressure gradients are eliminated by connecting the source and sink wells with additional channels and reservoirs that serve as low resistance pathways for fluid flow and pressure equilibration. Gradients can, thus, be maintained for several days and used to study cell migration in a sensitive and specific way with a variety of cell types [17]. The work presented here describes the use of such devices to assess chemotaxis for primary cultures of smooth muscle cells and to compare it with scratch and Boyden chamber techniques for sensitivity.

## 2. Materials and Methods

### 2.1. Primary Smooth Muscle Cells (SMC) Culture

 Aortas were harvested from 8-week-old C57/B6 mice (Charles River, Wilmington, MA) with sterile dissecting scissors. Adherent fat was removed, and aortas were incubated for 20 min at 37°C in Dulbecco's modified Eagle's medium (DMEM; Invitrogen, Grand Island, NY) containing 1% penicillin/streptomycin, 2% fetal bovine serum, and 5 mg/mL collagenase type II (Invitrogen). Aortas were rinsed in cold DMEM and the adventitia was carefully dissected away; they were then cut into small pieces and incubated for 30 min at 37°C in DMEM containing 1 mg/mL collagenase type I (Invitrogen) and 0.125 mg/mL elastase type III (Sigma-Aldrich, St.Louis, MO). Following repeated pipetting to dissociate the tissue, the resulting cell mixture was suspended in fresh “SMC medium” (DMEM with 10% fetal bovine serum, 1% penicillin/streptomycin; 2% nonessential amino acids, 1% L-glutamine; all reagents from Invitrogen) and grown at 37°C in a 5% CO_2_ incubator on plates coated with 1 mg/mL fibronectin for 30 minutes. Once cell culture is established (typically after the first passage), SMC are grown on uncoated plastic flasks (Corning Incorporated, Corning, NY).

SMC were used from passages 2 to 7 and were 99.5% pure as assessed by flow cytometry after staining for smooth muscle *α*-actin. For staining, cells are harvested and fixed with 1% paraformaldehyde (Sigma-Aldrich) in phosphate buffered saline (PBS). FITC-conjugated antismooth muscle *α*-actin antibody (Sigma-Aldrich) at 1 : 100 dilution in 10X BD perm/wash buffer (BD Pharmingen, San Jose, California) was applied for 10 min at room temperature. Cells are washed twice with 1% fetal bovine serum in PBS and once with 1% fetal bovine serum in PBS containing 1% formaldehyde and immediately analyzed by flow cytometry (FACScalibur, BD Biosciences, San Jose, CA). FITC-conjugated mouse IgG1 isotype (BD Pharmingen) is used as an isotype control. Cells for chemotactic assays were harvested when they had achieved confluence. 

### 2.2. Mitomycin-C Treatment

 Cells were trypsinized (0.25% trypsin-EDTA; Invitrogen) for 2 min at 37°C, washed with SMC medium, and then incubated as a single-cell suspension in 40 *μ*g/mL mitomycin-C (MMC; Sigma-Aldrich) in SMC medium for 30 min at 37°C. After two additional washes in PBS, cells were assessed for viability, proliferation, or migratory capacity. For the wound healing (scratch) assay, MMC treatment was performed after SMC plating and when cells were 100% confluent; cells were incubated in 40 *μ*g/mL MMC in SMC medium for 30 min at 37°C and then washed twice in PBS before assay.

### 2.3. SMC Proliferation Inhibition Assay

After MMC treatment or control incubation in SMC medium, SMC were cultured in 6-well plates (Corning Incorporated). Cells were recovered after 1 h, 24 h, or 48 h by trypsinization and cell numbers and viability were assessed by counting using a hemocytometer and trypan blue exclusion.

### 2.4. Wound Healing/Scratch Assay

SMC were cultured in 12-well plates (Corning Incorporated) until confluent and then treated with MMC. After washing, fresh SMC medium was added and the cell monolayer was scratched in a reproducible way using a sterile 200 *μ*L pipette tip. Different concentrations of platelet-derived growth factor (PDGF; R&D Systems, Minneapolis, MN) in SMC media were added to each well. The distance between the edges of the scratch defect were measured and averaged from five separate points at 3, 6, and 9 h, or until the wound defect had closed.

### 2.5. Boyden Chamber Transwell Assay

100 *μ*L of SMC suspensions at 1.0–1.5 × 10^5^ cells/mL (1.0–1.5 × 10^4^ total cells) were loaded into the upper transwell inserts (Costar, Corning Incorporated) and 600 *μ*L of SMC media containing different concentrations of PDGF were placed in the lower compartment. After 6 h, 12 h, or 24 h of incubation, the transwell membrane was stained with Hoechst 33342 (Invitrogen) according to the manufacturer's recommendation, and transmigrated cells on the lower face were counted on a fluorescence microscope using MetaMorph NX Microscopy Automation and Image Analysis Software (BioCompare, South San Francisco, CA). No cells were ever identified in the medium in the lower chamber. In experiments to assess whether cell migration represented chemotaxis or random chemokinesis in the absence of a concentration gradient, platelet derived growth factor-BB (PDGF) was added to the lower chamber, top insert well only, or both top insert and lower chamber.

### 2.6. Microfluidic Device Assay

The microfluidic devices (Figures 1(a)–1(c)) are prepared as described elsewhere [17]. As shown in Figure 1(d), gradients of added reagents are stable for at least 72 h. Depending on the experimental protocol, cells to be evaluated are placed in the central well and different chemotactic gradients developed from the two side wells. Alternatively, the migration of two different populations of cells can be evaluated from the side wells, experiencing identical concentration gradients generated by reagents placed in the central well. Cell concentrations were always 4-5 × 10^5^/mL and 40 *μ*L of the cell suspensions (1.6–2.0 × 10^3^ cells) was loaded into test wells.

**Figure 2:**
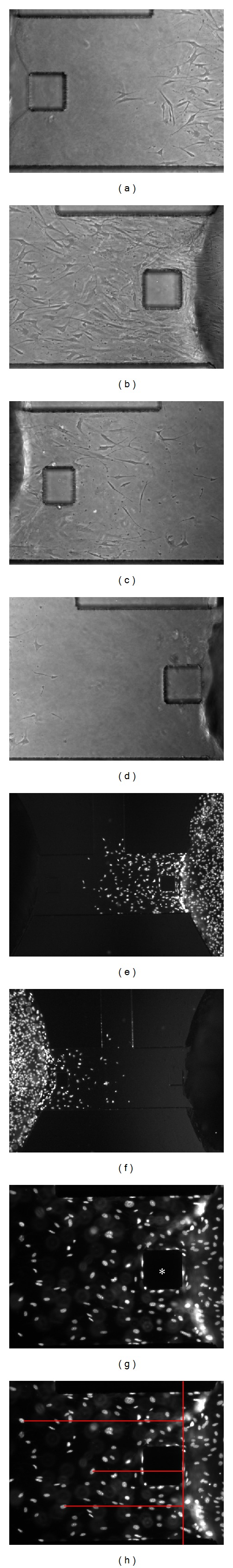
Assessment of cell migration in microfluidic devices. Cells were plated in the central lower well of a microfluidic device, with 50 ng/mL PDGF in SMC medium placed in the left lower well and SMC medium alone placed in the right lower well, followed by incubation at 37°C for 48 h. (a, b) Phase contrast image of cell chemotaxis from the central channel in the direction of the 50 ng/mL PDGF in the left channel; the images are split along the channel between the sink and source wells so that the full length of the channel can be shown at a magnification sufficient to permit cellular resolution. (c, d) Phase contrast image of cell chemokinesis from the central channel in the direction of SMC medium only. (e, f) Low-power fluorescence image of the left (e) or right; (f) channel at 48 h after staining with Hoechst 33342. (g) High-power fluorescence image of the left channel in panel (e); the black square without cells near the right-hand side of the image (asterisk) is a structural post that marks the start point for the migration. (h) Example of the migration analysis. A vertical red line denotes the start point; the distance from the start point to the center of each cell nucleus is measured, and the sum of all these individual measurements becomes the total migration distance using Matlab.

Cells were incubated in the devices for various time points and then stained with Hoechst 33342 (Invitrogen). The location of each cell was determined using fluorescence microscopy (Nikon Eclipse TE2000-U; Nikon Instruments, Inc., Melville, NY), and the distance migrated was assessed using the Matlab program (MathWorks, Natick, MA). “Total migration” is defined as the sum of the distances migrated by all cells beyond an initial starting location; this migration index is used for statistical analysis (Figure 2).

Collagen type I (Invitrogen) is used to fill the channel between central and side wells, and is the substrate through which cells migrate. Both 1 mg/mL and 2 mg/mL collagen can be used. Although 1 mg/mL collagen results in somewhat higher nonspecific background cell chemokinesis and gel loading is technically more difficult than with 2 mg/mL collagen, the lower collagen concentration permits greater migration of primary cultures of SMC, and the assay sensitivity is better. Unless otherwise specified, the concentration of collagen in the migration channels was 1 mg/mL for all experiments.

### 2.7. Statistical Analysis

Statistical comparisons were made using Student's *t*-test or one-way ANOVA. Significance was defined at the *P* < 0.05 level.

## 3. Results and Discussion

**Figure 3:**
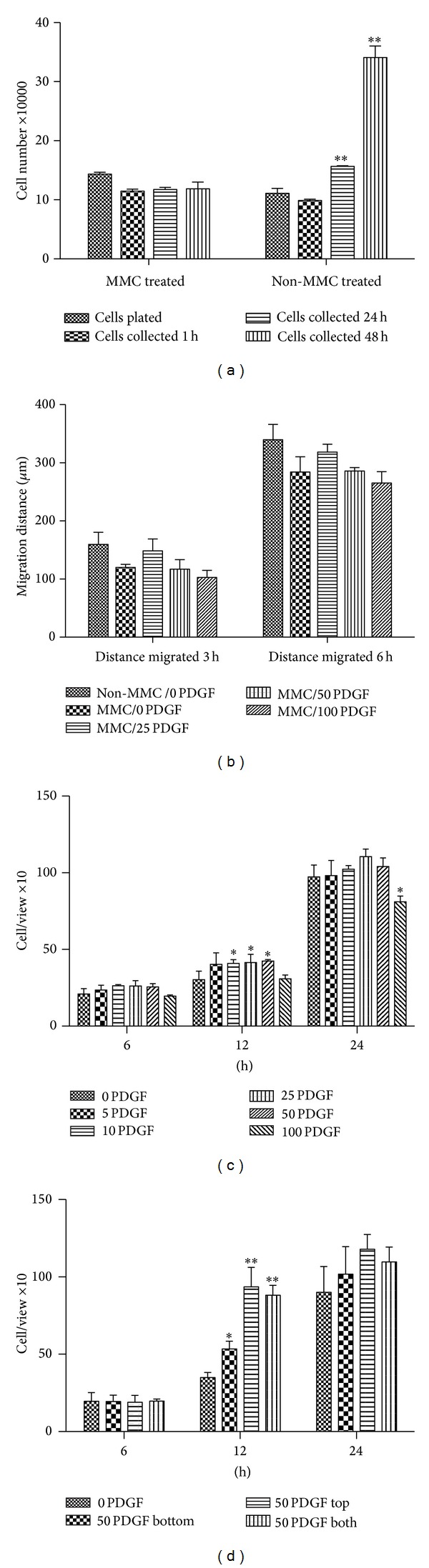
Cell migration in scratch and Boyden chamber assays. (a) *Mitomycin-C (MMC) inhibition of SMC proliferation*. SMC treated with or without MMC (40 *μ*g/mL for 30 min at 37°C) were plated in 6-well plates and subsequently harvested. Cell counts were manually performed on a hemocytometer after 1 h, 24 h, and 48 h; cell counts are expressed as the mean ± one standard deviation of triplicates. Trypan blue exclusion showed no differences in cell viability over 48 hr (91.8 ± 4.0%). MMC treatment leads to stable numbers of cells collected at 24 h and 48 h with significantly increased proliferation in the non-MMC-treated populations (**P < 0.01). (b) *Scratch assay*; no differences are seen in the extent of migration between cells cultured in no chemokine versus 25–100 ng/mL PDGF; over the 3–6 hours of this assay, no significant difference is seen between control cells and those treated with MMC. (c) *Transwell assay*; different concentrations of PDGF were added to the lower chamber of transwell devices at time zero; cells on the lower aspect of the transwell insert were enumerated after 6 h, 12 h, or 24 h. Compared with no PDGF in the lower chamber, there were significantly greater cell numbers of migrated cells in 10 ng/mL, 25 ng/mL, and 50 ng/mL PDGF group after 12 h (*P < 0.05). After 24 hours, migrated cell numbers in transwells with 5–50 ng/mL PDGF were not significantly different relative to chambers without PDGF. After 24 hours, migrated cell numbers when 100 ng/mL PDGF was present in the lower chamber were significantly reduced relative to the control 0 ng/mL PDGF group. (d) *Chemokinesis in transwells*; the same 50 ng/mL concentration of PDGF was loaded into bottom chamber, top chamber, or both bottom and top chambers; cell migration was analyzed after 6 h, 12 h, or 24 h. Relative to no PDGF in either chamber, cell migration was significantly greater with 50 ng/mL in the lower chamber at 12 hours (*P < 0.05); by 24 hours, there was no significant difference between control chambers and 50 ng/mL PDGF. Notably, adding PDGF to the upper chamber, with or without PDGF in the lower chamber, led to significantly increased migration at 12 hours relative to PDGF in the lower chamber alone (*P < 0.05).

As shown in Figure 3(a), cell proliferation is blocked by MMC treatment. There is no significant difference in cell viability between MMC-treated SMC (91.8 ± 4.0%) and untreated SMC (93.0 ± 4.4%) for up to 48 hours after treatment. Thus, cell accumulation in the various migration assays represents true cell movement without any component of cell proliferation. This is important because without MMC treatment, migration indices from long-term incubations may be confounded by coincident cellular proliferation.

In scratch assays (Figure 3(b)) with primary SMC cultures, cells randomly migrated (chemokinesis) to fill in the defects at comparable rates regardless of PDGF concentration, including in the absence of any chemotactic agent. Without MMC treatment, wound defects tend to close slightly earlier, suggesting an element of cell proliferation.

Figure 3(c) shows the migration results for SMC using the Boyden chamber transwell assay. The early (12 h) time point shows small but significantly increased migration in response to 10–50 ng/mL PDGF versus no chemokine in the bottom well. However, there was no distinguishable difference in migration over a 10 fold concentration range of PDGF, suggesting that the assay is relatively insensitive, at least for primary SMC cultures. Moreover, by 24 hours, there are no differences among any of the concentrations of chemokines (including the medium control) suggesting that migration at this time point is nonspecific once the cytokine concentrations have begun to equilibrate between the top and bottom wells.

To formally demonstrate that migration across the membrane in the upper well is not necessarily due to directed chemotaxis, PDGF was added to the top well, with or without PDGF in the lower compartment. Even in the absence of a chemokine gradient, PDGF in the upper chamber led to markedly increased transmigration (Figure 3(d)); this can only be attributed to increased chemokinesis.

The transwell experiments, thus, suggest that although initial PDGF concentration differences can induce some degree of increased cell migration towards higher concentrations in the lower wells, subsequent PDGF diffusion leads to random chemokinesis.

**Figure 4:**
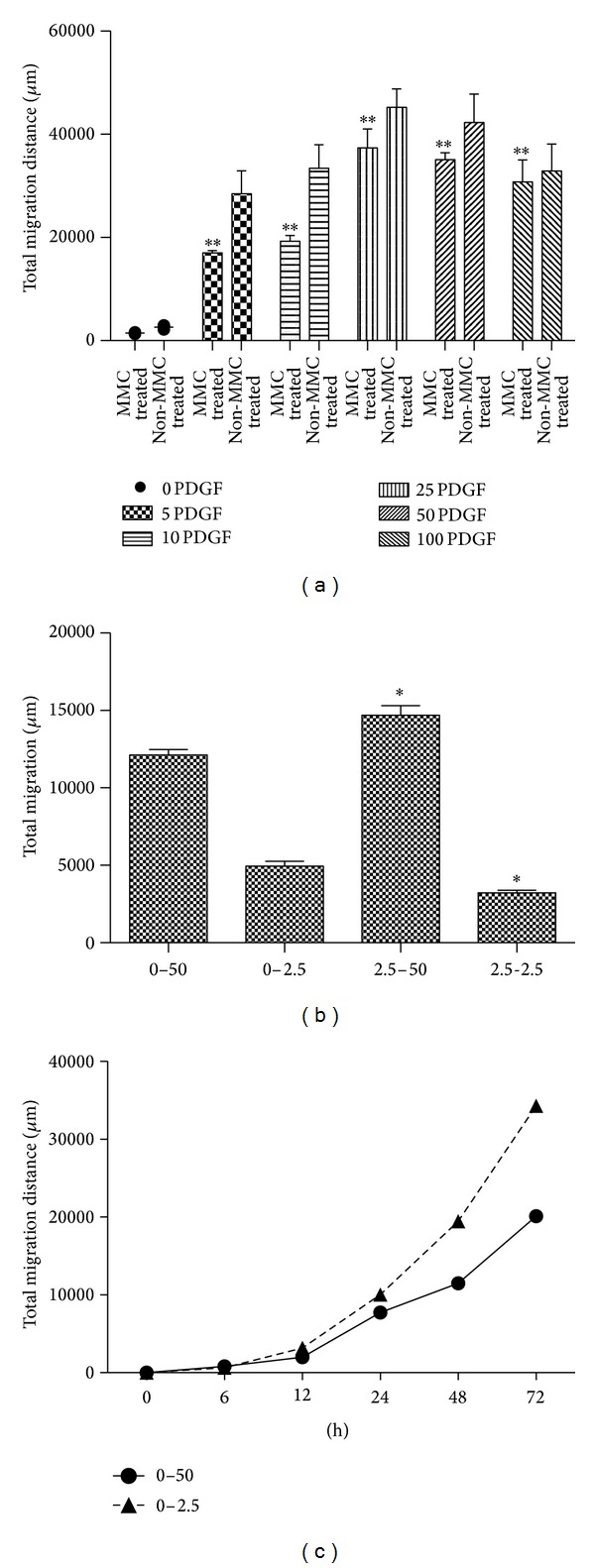
Microfluidic device assay. (a) *Dose-response curves with and without prior MMC treatment*. MMC-treated and nontreated SMC were placed in side cell wells, with different concentrations of PDGF (5 ng/mL, 10 ng/mL, 25 ng/mL, 50 ng/mL, and 100 ng/mL) present in the central source wells. Migration was measured after Hoechst 33342 staining and fluorescence microscopy after 48 h, using the Matlab program to calculate total migration. Controls (SMC medium only) and every concentration of PDGF were evaluated in triplicate devices. Significant differences can be seen from 5 ng/mL to 100 ng/mL PDGF compared with the control (**P < 0.01). (b) *Chemokinesis versus chemotaxis in microfluidic devices*. MMC-treated SMC with or without 2.5 ng/mL PDGF were plated in the central cell well; 2.5 ng/mL or 50 ng/mL PDGF were added to the side source wells. With no PDGF present in the central cell well, the assay shows significantly greater total migration after 48 h with 50 ng/mL in the source well (“0–50”) *versus* 2.5 ng/mL in the source well (“0–2.5”). The presence of 2.5 ng/mL PDGF in the central cell well leads to significantly greater total migration when there is a concentration gradient (“2.5–50”; *P < 0.05) attributable to a local chemokinesis effect. In the absence of a gradient (“2.5–2.5”), there is a chemokinesis-associated migration which is significantly less than the migration seen when a gradient is present (“0–2.5”; *P < 0.05). (c) Time-course experiment performed as panel (b).

In comparison, dose-response experiments using the microfluidic devices (Figure 4) demonstrate significant chemotaxis at concentrations as low as 2.5 ng/mL PDGF (Figure 4(b)), and show a dose-dependent increase in chemotaxis with a peak of approximately 25–50 ng/mL (Figure 4(a)). Interestingly, higher concentrations of PDGF (e.g., 100 ng/mL) lead to less migration, consistent with a high-dose chemotaxis arrest (18). Of note, without prior MMC treatment, the migration index is greater (Figure 4(a)), highlighting the nonnegligible contribution of proliferation to “apparent” chemotaxis occurring with longer assay times. The results demonstrate that the microfluidic device for measuring chemotaxis is substantially more sensitive than the existing scratch or “gold standard” Boyden chamber approaches.

Not only can the microfluidic device be used for measuring chemotaxis, but also it can specifically distinguish the contributions of chemokinesis and chemotaxis to migration (Figure 4(b)). Thus, in the absence of a chemokine gradient (e.g., 2.5 ng/mL PDGF in both center and side wells), the migration index reflects chemokinesis. In comparison, with no PDGF in the center well and 2.5 ng/mL in the side well, the migration index reflects directed chemotaxis. The microfluidic device can also assess chemotaxis in the presence of existing chemokinesis, as when the center well contains 2.5 ng/mL PDGF and the side well contains 50 ng/mL. There is a small but statistically greater migration when the stimulus is a chemotactic gradient rather than simple chemokinesis.

Since the chemokine gradient is established within 2 hours (17), and is stable for several days, cells can continuously migrate up the concentration gradient over time (Figure 4(c)). In comparison, other classic methods either have no concentration gradient (scratch assay) or only have a transient chemokine gradient, so that chemokinesis—rather than directed chemotaxis—is increasingly contributory.

The microfluidic device reported in this work is superior in a number of respects compared with other *in vitro* approaches previously used to study chemotaxis. In particular, the Boyden chamber—involving a chemokine gradient across a porous membrane—has been a standard chemotactic assay since the 1950s. However, this device has significant limitations in that gradients are neither stable nor linear, with an initial sharp concentration step up that progressively deteriorates over time. More recently micro-electromechanical systems (MEMS) technologies were developed that utilize microfluidic biochips to mimic *in vivo* conditions; in these devices, cells are continually exposed to shear forces under controlled flow. However, the continuous flow of these devices presents a challenge to the maintenance of stable chemokine gradients. Although hydrogels between the source and sink channel can create linear concentration gradients [18–20], subtle variations in the wells and channels can engender pressure gradients that disrupt the gradients through interstitial flow [21, 22]; moreover, the continuous flow of the devices tends to dilute any chemoattractants secreted by cell sources. In the novel microfluidic device described previously [17] and validated for smooth muscle cell migration in this paper, large volume source and sink wells maintain stable chemokine gradients in the connecting hydrogel; any interstitial flow is eliminated by connecting the source and sink wells with additional channels and reservoirs that serve as a resistor-capacitor circuit. The concentration gradients are, thus, stable and linear for several days. The current work has further refined the application, incorporating mitomycin-C treatment to reduce any confounding contribution of proliferation and demonstrating how chemotaxis and chemokinesis can be assessed in the same experimental setup.
